# Inhibition of litter decomposition of two emergent macrophytes by addition of aromatic plant powder

**DOI:** 10.1038/s41598-017-16615-8

**Published:** 2017-11-30

**Authors:** Ya-jun Xie, Yong-hong Xie, Hua-yun Xiao, Zheng-miao Deng, Ying Pan, Bai-han Pan, Jia-yu Hu

**Affiliations:** 1 Jiangxi Province Key Laboratory of the Causes and Control of Atmospheric Pollution, East China University of Technology, Nanchang, 330013 China; 2 School of Water Resources and Environmental Engineering, East China University of Technology, Nanchang, 330013 China; 3 Dongting Lake Station for Wetland Ecosystem Research, Key Laboratory of Agro-ecological Processes in Subtropical Regions, Institute of Subtropical Agriculture, The Chinese Academy of Sciences, Changsha, 410125 China

## Abstract

Aromatic plants show antimicrobial activity due to their essential oils, but their effect on litter decomposition is unclear. In this study, we evaluated the biomass loss and nutrient dynamics in leaf litters of two macrophytes (*Miscanthus sacchariflorus* and *Carex brevicuspis*) with and without addition of powdered material of the aromatic plant *Polygonum hydropiper* or the non-aromatic plant *C. brevicuspis*. The two powders had similar basic chemical qualities but *P. hydropiperi* had a higher essential oils concentration. Leaf litters of *M. sacchariflorus* and *C. brevicuspis* were incubated with powdered *P. hydropiper* or *C. brevicuspis* (500 g m^−3^, 250 g m^−3^, and no addition) for 120 days in a mesocosm experiment. Compared with the control (no addition), *P. hydropiperi* addition decelerated nutrient release and litter decomposition, while *C. brevicuspis* addition accelerated those processes. The nitrogen concentrations in both leaf litters and the phosphorus concentration in *C. brevicuspis* leaf litter were increased by addition of both plant powders. The fungal biomass in both leaf litters decreased after *P. hydropiperi* addition, due to the antifungal activity of its essential oils. These data indicate that the aromatic plant *P. hydropiperi* inhibits litter decomposition via its essential oils and that such inhibition is not species-specific.

## Introduction

Litter decomposition partly controls the rates of nutrient and carbon cycles^1^. The rate of litter decomposition is regulated by interacting physical, chemical, and biotic factors, such as climate (especially temperature and moisture), initial litter quality variables (e.g. nitrogen and phosphorus concentrations, lignin concentration, and carbon:N ratio), exogenous nutrient availability, and decomposers (microbes and invertebrates)^2,3^. Plants create different environments that retard or accelerate litter decomposition through negative or positive effects on the activity of organisms^4^.

Aromatic plants are those that synthesize and emit essential oils. These oils are insoluble and include various groups, for example, terpenes, alcohols, and aldehydes^5,6^. Aromatic plants are widely used in medicine and food storage, and their essential oils have been shown to interfere with the growth and enzymatic reactions of microbes *in vivo* and *in vitro* ^7,8^. In the Mediterranean region, fallen litter or living parts of aromatic plants release essential oils that remain in the soil for up to 1 year^6,9^. Because aromatic plants always coexist with other plant species, they might affect the decomposition of litter via the effects of their essential oils on microbes^6^. Besides essential oils, nutrients released from aromatic plants may accelerate the decomposition of other litters^10^. However, the effects of aromatic plants on litter decomposition remain unclear.

*Polygonum hydropiper*, a common aromatic plant in East Asia, is famous for its antimicrobial activity and is used as a pharmaceutical^11,12^. Previous studies have reported on its secondary compounds^8^, among which its essential oils (mainly terpenes) are reported to affect microbes^12^. The objective of this study was to investigate the overall effect of *P. hydropiper* on the decomposition of leaf litter of *Miscanthus sacchariflorus* and *Carex brevicuspis*, two dominant plants at Dongting Lake, the second largest freshwater lake in China. In the Dongting Lake wetlands, *P. hydropiper* often coexists with *C. brevicuspis* but seldom with *M. sacchariflorus*. Leaf litters from the two plants were incubated with and without powdered plant materials of *P. hydropiper* and *C. brevicuspis*. The two powders had similar basic chemical qualities, but *P. hydropiperi* had a higher essential oils concentration (Table 1). We tested the following hypotheses: first, that *C. brevicuspis* litter would decompose faster than *M. sacchariflorus* litter because of the high nutrient concentrations in *C. brevicuspis*; second, that the decomposition rates of both litters would be increased by *C. brevicuspis* addition due to the addition of exogenous nutrients; third, that the decomposition rates of both litters would be decreased by *P. hydropiper* addition due to its essential oils.

**Table 1:** Basic properties of soil substrate at the beginning of incubation and qualities of plant powders.

Material	Organic C concentration (%)	N concentration (%)	P concentration (%)	Essential oils concentration (% dry mass)
**Plant powder species**				
*P. hydropiper*	42.21 ± 2.11	2.07 ± 0.103	0.362 ± 0.051	0.42 ± 0.11**
*C. brevicuspis*	44.14 ± 1.88	1.91 ± 0.142	0.225 ± 0.066	0.01 ± 0.00
**Soil type**				
No addition	1.73 ± 0.14	0.110 ± 0.004 a	0.060 ± 0.002 a	—
Low *P. hydropiper*	1.71 ± 0.06	0.120 ± 0.003 b	0.065 ± 0.001 b	—
High *P. hydropiper*	1.71 ± 0.10	0.129 ± 0.002 c	0.063 ± 0.002 b	—
Low *C. brevicuspis*	1.73 ± 0.07	0.121 ± 0.005 b	0.061 ± 0.002 ab	—
High *C. brevicuspis*	1.73 ± 0.07	0.131 ± 0.005 c	0.063 ± 0.001 b	—

Values are means ± S.E. (*n* = 3). Different lowercase letters (a, b, c) indicate significant difference in soil substrate among treatments (LSD test, *P* < 0.05). **Indicates significant difference between *P. hydropiper* and *C. brevicuspis* powders (*t*-test, *P* < 0.01).

## Results

### Initial litter quality

The initial N, P, organic C, cellulose, and lignin concentrations differed significantly between the two litters (*P* < 0.05; Table 2). The concentrations of both N and P were higher in *C. brevicuspis* litter than in *M. sacchariflorus* litter (*P* < 0.05), while the initial lignin and cellulose concentrations, and C:N, C:P, and lignin:N were higher in *M. sacchariflorus* litter than in *C. brevicuspis* litter (*P* < 0.05). These results suggested that *C. brevicuspis* litter may have greater decomposition potential than *M. sacchariflorus* litter.

**Table 2:** Initial quality of two species of plant litter.

Parameter	Litter species		Significance
	*M. sacchariflorus*	*C. brevicuspis*	
N concentration (%)	0.415 ± 0.085	0.768 ± 0.018	**
P concentration (%)	0.048 ± 0.013	0.089 ± 0.010	**
Organic C concentration (%)	43.13 ± 0.85	38.37 ± 1.77	**
Cellulose concentration (%)	18.56 ± 2.53	14.61 ± 0.31	**
Lignin concentration (%)	37.82 ± 1.06	35.88 ± 1.66	*
C:N ratio (molar molar^−1^)	125.69 ± 31.85	58.36 ± 3.80	**
C:P ratio (molar molar^−1^)	1100.71 ± 269.17	506.18 ± 32.49	**
N:P ratio (molar molar^−1^)	10.35 ± 2.26	10.17 ± 1.27	ns
Lignin:N ratio (molar molar^−1^)	94.49 ± 24.22	46.75 ± 2.44	**

Values are means ± S.E. (*n* = 3). *P < 0.05; **P < 0.01; ns, no significant difference.

### Litter decomposition

**Figure 1:**
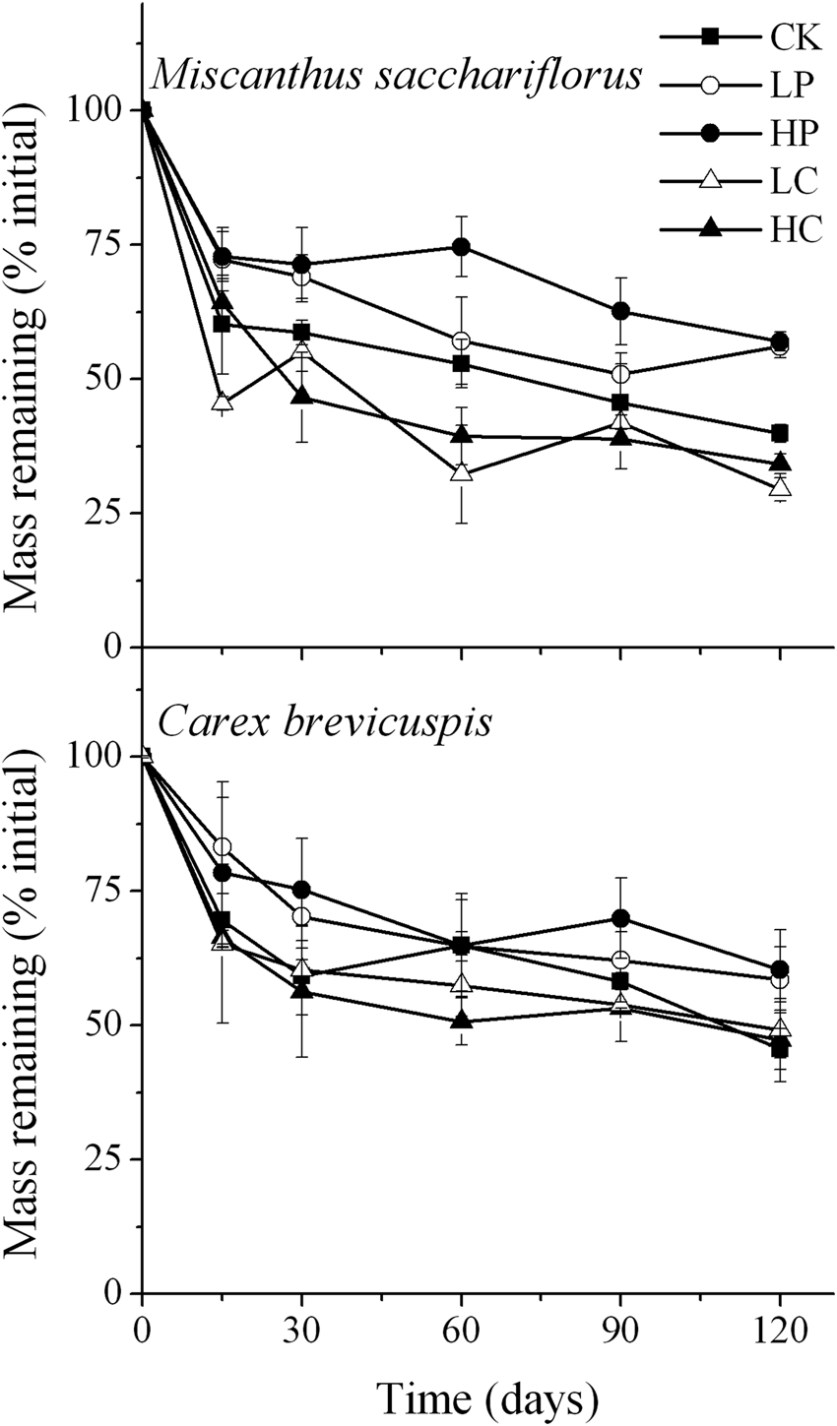
Percentage of mass remaining in two litter species in five plant addition treatments. Values are means ± S.E. (*n* = 3). CK, no addition; LP, low addition of *P. hydropiper*; HP, high addition of *P. hydropiper*; LC, low addition of *C. brevicuspis*; HC, high addition of *C. brevicuspis*.

In all treatments, the litters decayed most quickly in the initial 2 weeks of the experiment, and more slowly thereafter (Fig. 1). Within the same treatment, the *M. sacchariflorus* litter decomposed faster than did *C. brevicuspis* litter (three-way ANOVA, F = 64.35, *P* < 0.01; Table 3). The fastest decomposition was in *M. sacchariflorus* litter with powdered *C. brevicuspis* at 500 g m^−3^, and the slowest decomposition was in *C. brevicuspis* litter with powered *P. hydropiper* at 500 g m^−3^. Two-way ANOVA showed significant effects of powdered plant addition on the decomposition rates of both litter species (*P* < 0.01; Table 4). Compared with the control, *P. hydropiper* addition at 250 g m^−3^ decreased the decomposition rates of *M. sacchariflorus* and *C. brevicuspis* litter by 26% and 17%, respectively, and *P. hydropiper* addition at 500 g m^−3^ decreased the decomposition rates of *M. sacchariflorus* and *C. brevicuspis* litter by 41% and 31%, respectively (*P < *0.01; Tables 3 and 4). However, the decomposition rates of *M. sacchariflorus* and *C. brevicuspis* litter increased after the addition of powdered *C. brevicuspis* (*P* < 0.01; Tables 3 and 4).

**Table 3:** Regression statistics (*R* ^2^) for exponential rates of decomposition (*k*, unit g g^−1^ dry mass day^−1^).

Treatments	*M. sacchariflorus* litter		*C. brevicuspis* litter	
	*k*	*R* ^2^	*k*	*R* ^2^
No addition	0.0061 ± 0.0008	0.791	0.0044 ± 0.0006	0.700
Low *P. hydropiper*	0.0045 ± 0.0007	0.726	0.0040 ± 0.0005	0.821
High *P. hydropiper*	0.0036 ± 0.0004	0.744	0.0033 ± 0.0003	0.742
Low *C. brevicuspis*	0.0076 ± 0.0007	0.756	0.0045 ± 0.0006	0.650
High *C. brevicuspis*	0.0075 ± 0.0008	0.628	0.0048 ± 0.0005	0.690

Values are means of three replicates ± S.E. (*n* = 3).

**Table 4:** Two-way ANOVA results for mass, N remaining, P remaining, and N and P concentrations in two litter species in three plant addition (PA) treatments.

Parameter	Source of variation	*P. hydropiper* addition		*C. brevicuspis* addition	
		*M. sacchariflorus* litter	*C. brevicuspis* litter	*M. sacchariflorus* litter	*C. brevicuspis* litter
		*F* Value	*F* Value	*F* Value	*F* Value
Mass	Time	18.499**	10.168**	26.712**	8.961 ns
	PA	33.878**	7.737**	15.328**	2.663*
	Time × PA	1.642 ns	0.887 ns	3.731**	0.638 ns
N remaining	Time	6.767**	4.513**	10.587**	2.525 ns
	PA	63.862**	18.444**	4.882*	0.040 ns
	Time × PA	1.983 ns	3.493**	5.053**	2.101 ns
P remaining	Time	3.957*	10.437**	10.969**	4.253**
	PA	13.280**	4.421*	14.649**	0.032 ns
	Time × PA	1.297 ns	0.461 ns	2.965*	1.677 ns
N concentration	Time	33.930**	24.367**	72.653**	28.797**
	PA	20.278**	13.603**	4.180*	4.908*
	Time × PA	3.848**	4.877**	2.740*	4.015**
P concentration	Time	7.688**	4.668**	15.547**	0.381 ns
	PA	0.756 ns	2.811*	0.899 ns	2.729*
	Time × PA	1.018 ns	2.053 ns	0.699 ns	2.856*

*P < 0.05; **P < 0.01; ns, no significant difference.

**Figure 2:**
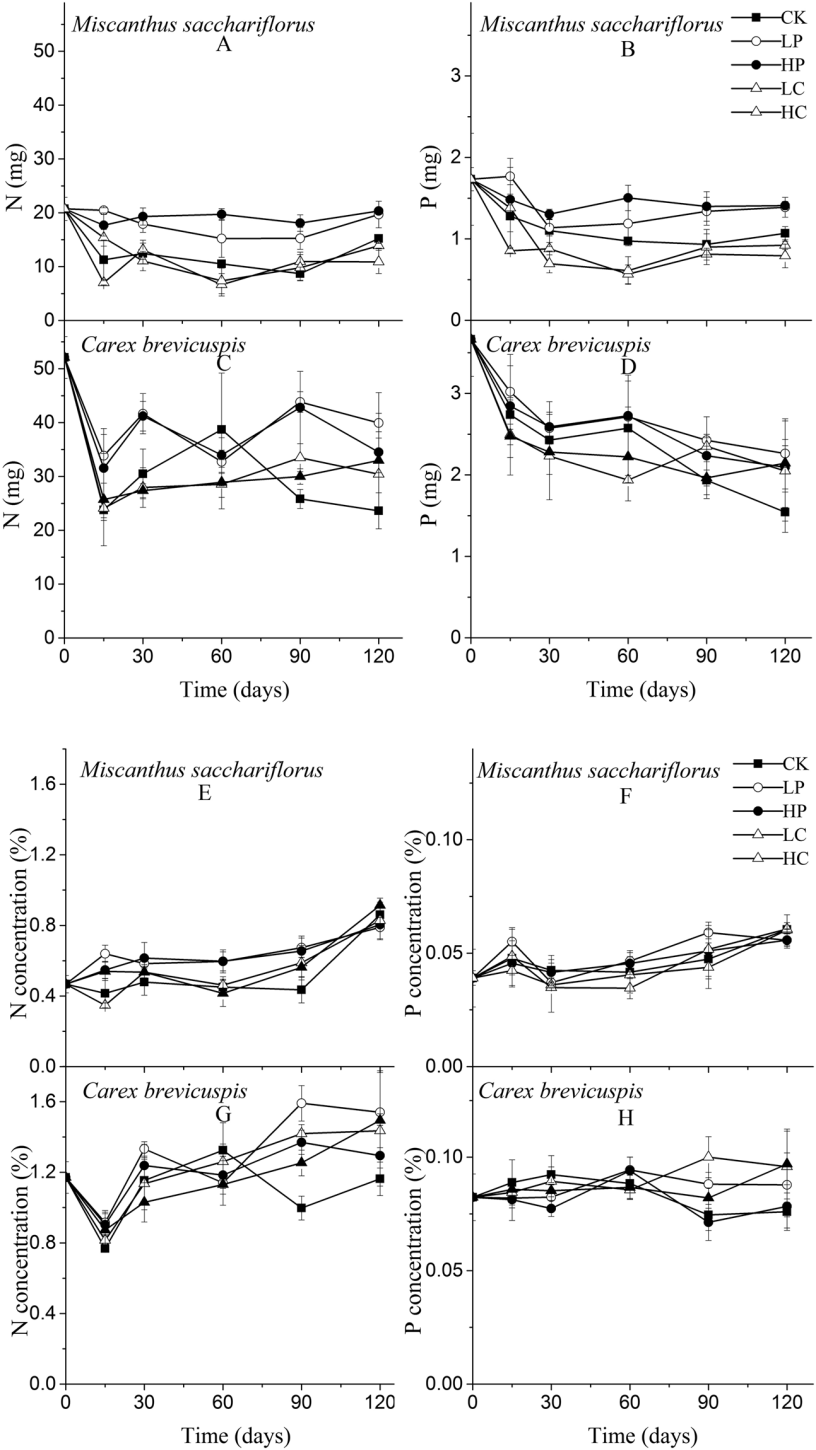
Nutrients contents (mg) and concentrations (%) remaining in two litter species in five plant addition (PA) treatments. Values are means ± S.E. (*n* = 3). CK, no addition; LP, low addition of *P. hydropiper*; HP, high addition of *P. hydropiper*; LC, low addition of *C. brevicuspis*; HC, high addition of *C. brevicuspis*.

The N content decreased initially in both species but then recovered (Fig. 2A,C). The N concentration in *M. sacchariflorus* litter increased throughout the incubation period, while N concentration in *C. brevicuspis* initially declined, then increased (Fig. 2E,G). Similarly, the P content decreased initially (*M. sacchariflorus* litter) or throughout the incubation period (*C. brevicuspis* litter) (Fig. 2B,D). The total P concentration increased steadily (*M. sacchariflorus* litter) or remained approximately constant (*C. brevicuspis* litter) (Fig. 2F,H). The release of N and P from both leaf litters was inhibited by *P. hydropiper* addition (*P* < 0.01; Table 4), while *C. brevicuspis* inhibited P and N release only from *M. sacchariflorus* leaf litter (*P* < 0.01; Table 4).

The addition of the powdered plant materials resulted in significant nutrient enrichment of both leaf litters, as indicated by the N and P concentrations (Fig. 2E–H). Addition of powdered *P. hydropiper* and *C. brevicuspis* increased the N concentrations in both *M. sacchariflorus* litter and *C. brevicuspis* litter as well as the P concentration in *C. brevicuspis* litter (*P* < 0.01; Table 4).

There were significant interactions between time and plant addition for nutrient dynamics (*P* < 0.01; Table 4), indicating that the effects of plant addition mainly occurred at the later stage of the incubation period.

### Fungal biomass

**Figure 3:**
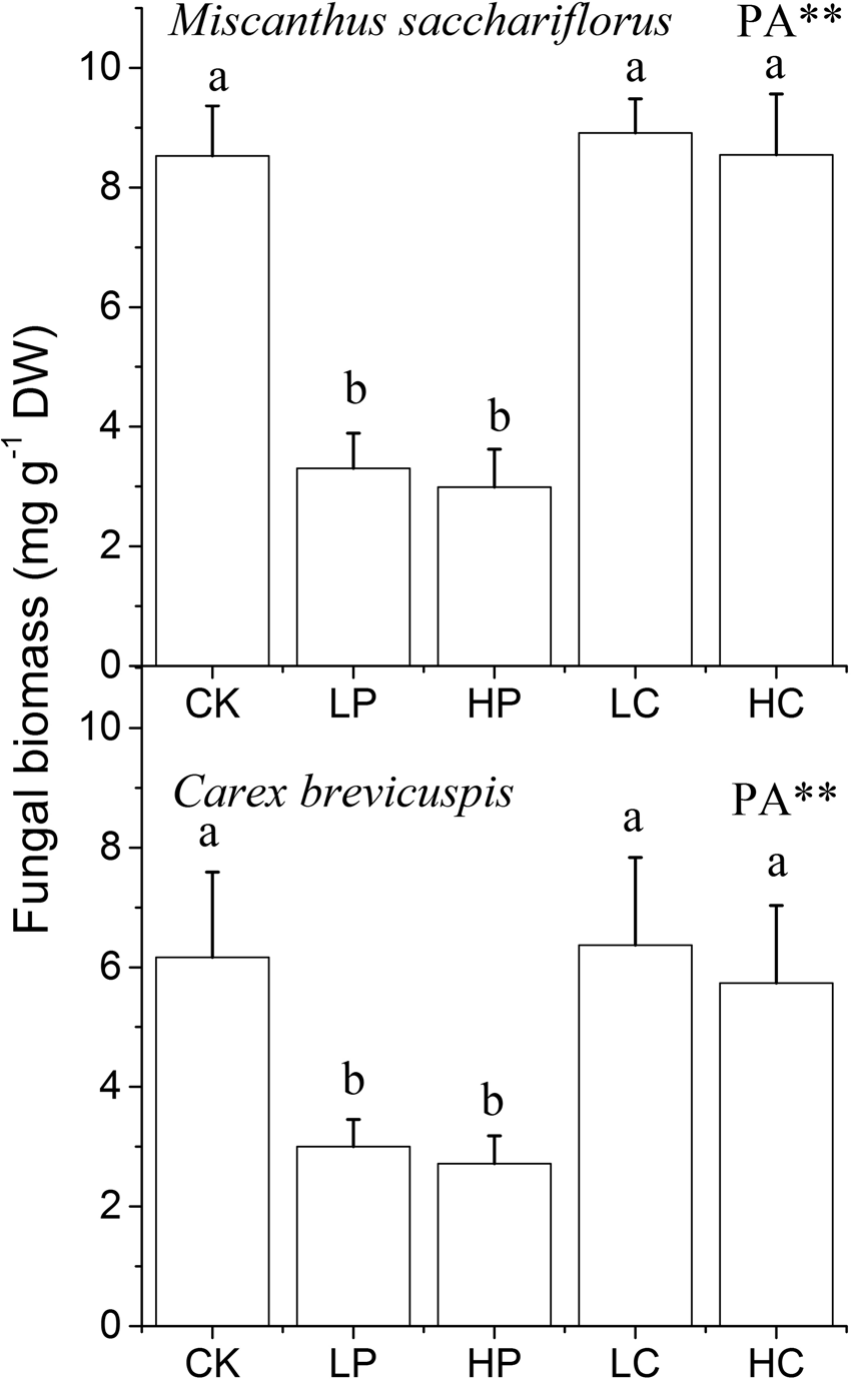
Fungal biomass in two litters with five plant addition (PA) treatments. Values are mean ± S.E. (*n* = 3). CK, no addition; LP, low addition of *P. hydropiper*; HP, high addition of *P. hydropiper*; LC, low addition of *C. brevicuspis*; HC, high addition of *C. brevicuspis*. **P < 0.01. Different lower case (a, b) letters indicate significant difference in fungal biomass among treatments.

The fungal biomass was higher in *M. sacchariflorus* litter than in *C. brevicuspis* litter in the treatments with and without powdered *C. brevicuspis* addition (*P* < 0.05; Fig. 3). The fungal biomass did not differ significantly between *M. sacchariflorus* litter and *C. brevicuspis* litter in the treatments with *P. hydropiper* addition (*P* > 0.05). At the end of the experiment, the fungal biomass in *M. sacchariflorus* litter and *C. brevicuspis* litter was lower in the treatments with powdered *P. hydropiper* (*P* < 0.05; Fig. 3) than in the treatments with powdered *C. brevicuspis* (*P* > 0.05; Fig. 3).

## Discussion

Within the same treatment, *M. sacchariflorus* litter decomposed faster than *C. brevicuspis* litter, indicating that litter nutrient concentration was not the sole determinant of the decomposition rate of these aquatic macrophytes^13^. Other qualities, such as the tenderness of *M. sacchariflorus* leaves, can result in faster decay^14,15^.

Decomposition of both *M. sacchariflorus* and *C. brevicuspis* litter was enhanced by addition of powdered *C. brevicuspis*, as proposed in the second hypothesis. This increased decomposition may result from nutrient enrichment due to plant addition, as reported previously^16^. Generally, litter decomposition is the process of utilization by decomposers^17^. Fungal biomass in our study was similar with previous result^18^. Though their biomass was not high, fungi were still the fundamental decomposers over the bacteria in wetland environments^18^. Fungi must assimilate available nutrients from their environment, so nutrient availability is an important limiting factor in litter decomposition^17^. In previous studies, a positive correlation between mass loss and initial litter nutrient concentrations suggested that litter N and P concentrations could predict the litter decomposition rate^19,20^. It is worth mentioning that adding materials in powdered form would increase the availability of N and P, because the physical structure would be destroyed and there would be a large surface area for microbial attack. In this study, the addition of powdered *C. brevicuspis* increased the N concentration in both leaf litters and the P concentration in *C. brevicuspis* litter, suggesting that nutrients might be transported from *C. brevicuspis* powder to the leaf litters. Such nutrient enrichment might meet the nutrient demands of fungi, although fungal biomass was not affected by *C. brevicuspis* addition.

Litter decomposition was accelerated by addition of powdered *C. brevicuspis*, but inhibited by addition of powdered *P. hydropiper*, indicating that aromatic plant material inhibited litter decomposition, consistent with the third hypothesis. The responses of both leaf litters were similar, suggesting that the inhibition of litter decomposition by addition of *P. hydropiper* might not be species-specific.

A previous study reported that addition of the green leaves of the aromatic plant *Alliaria petiolata* promoted litter decomposition^21^. The authors of that study proposed that the positive effects of nutrient enrichment from *A. petiolata* on decomposition outweighed any negative effects of secondary compounds on the activity of the microbes decomposing the litter. In our study, *P. hydropiperi* addition did not stimulate litter decomposition. Given the difference in chemical qualities between the two powders, the abundant essential oils in *P. hydropiperi* was the most probable reason for our results. In the present study, the nutrient concentrations in litter were enriched by adding powdered *P. hydropiper*, but these nutrients may have been retained in the litter instead of being released. This is consistent with the decreases in nutrient release after *P. hydropiper* addition reported in other studies^22,23^. Such nutrient retention may be related to the physical effect of essential oils; that is, the hydrophobicity of essential oils coating the litter could prevent microbes from acquiring nutrients^24^. If this was the case, then it would have been difficult for microbes to access and utilize retained nutrients after the addition of powdered *P. hydropiper*. Another explanation may be the antifungal activity of essential oils, as indicated by the decreases in fungal biomass in both litters after *P. hydropiper* addition. The antifungal activity of *P. hydropiper* essential oils has been reported previously^8,11^. Given the fundamental role of fungi in litter consumption, this inhibition of fungal activity by *P. hydropiper* addition might lead to a decreased litter decomposition rate.

In this study, the litter decomposition rates decreased after addition of aromatic plant material. Therefore, in previous studies, the biogeochemical cycle rates might be overestimated for some ecosystems containing aromatic plants. Studies on the return rates of C and other nutrients to the ecosystem should take the presence of aromatic plants into account. In this study, we focused only on the aromatic plant *P. hydropiper*. Further studies are required to determine the effects of other aromatic plant materials on litter decomposition.

## Conclusions

Our findings provide insights into the mechanisms by which aromatic plants affect litter decomposition rates and nutrient dynamics in wetlands. Our findings showed that litter decomposition was stimulated by addition of powdered material from a non-aromatic plant, but inhibited by addition of powdered material from an aromatic plant, probably by essential oils, and such inhibition is not species-specific.

## Methods

### Collection and preparation of plant material and soil

Leaf litters of *M. sacchariflorus* and *C. brevicuspis* were collected from standing dead plants. Aerial parts of *P. hydropiper* and *C. brevicuspis* were collected from living plants at Dongting Lake (29°27′2″N, 112°47′32″E) in November 2012. Aerial parts were selected because the essential oils concentration seldom changed during withering^6^. *M. sacchariflorus*, *C. brevicuspis*, and *P. hydropiper* are the main emergent macrophytes in these wetlands, with biomasses of 3000 g m^−2^, 1500 g m^−2^, and 2500 g m^−2^, respectively. Control soil was collected from the 0–15 cm layer from bare land nearby to avoid the potential home-field advantage during decomposition^25^. This soil was loam silt and its basic properties are shown in Table 1 (no addition). After collection, the leaf litters were air-dried to constant mass for 48 h and cut into approximately 10-cm long pieces. Weighed litter samples (5 g) were placed into 10 × 15-cm nylon bags (1-mm mesh). This mesh size excluded macroinvertebrates but allowed microbial colonization and leaching of litter fragments^26^. Sets (‘strings’) of two bags (one per litter species) were connected by nylon string to facilitate harvest. The aerial part was oven-dried at 60 °C for 7 days to avoid loss of essential oils, and then ground to a powder and passed through a 0.5-mm mesh screen^27^. Reduction of the added material to a powder would render the N and P in it readily available because the physical structure would be destroyed and there would be many surfaces for microbial attack.

### Experimental set-up

The study was carried out at the Dongting Lake Station for Wetland Ecosystem Research (29°29′59″N, 112°47′49″E). This site has a subtropical monsoon climate. The average annual air temperature is 17 °C and the average annual precipitation is 1,302 mm.

The mesocosm experiment consisted of the soil substrate with and without added plant powder^28^. The experimental treatments consisted of two leaf litters without (control, CK) or with addition of powdered plant materials of *P. hydropiper* and *C. brevicuspis* at two levels (high addition, 500 g m^−3^; low addition, 250 g m^−3^) in a one-way factorial design in 15 plastic tanks (0.5 × 0.4 × 0.7 m; five treatments, each with three replicates). The high addition rate was selected based on the biomass (2500 g m^−2^) of *P. hydropiper* in the Dongting Lake wetlands. To simulate the soil environment under an aromatic plant, the fermentation system allowed materials such as nutrients and essential oils to be released from plant powder into the soil substrate and then to contact the litter. Each tank was filled with soil to a depth of 0.2 m. After homogenizing, the soil substrate was saturated with water and fermented for 30 days to reduce the potential effects of new organic C input^29^ on litter decomposition. Besides, since the powder decomposed substantially after fermentation (see the organic C concentration of various soil substrate in Table 1), the potential non-additive effects of litter mixing was also prevented. The addition of plant powder increased the nutrient concentrations in all soil substrates (*P* < 0.05; Table 1) except for P in the 250 g m^−3^ *C. brevicuspis* treatment. The soil organic C concentration was not affected by plant addition (*P* > 0.05; Table 1).

Litter strings were randomly placed in the tanks (at 5-cm intervals) on April 14, 2013, and buried to 5-cm depth in the substrate. A total of 180 litterbags were used (three replicates × two litter species × five treatments × five harvests). During the incubation period, tap water was added to each tank weekly to maintain soil water at approximately 60% of water holding capacity for optimal microbial activity^30^. Three strings per treatment were sampled after incubation for 15, 30, 60, 90, and 120 days. Another 30 samples (three replicates of two litter species and five treatments) were sampled to measure the ergosterol concentration on day 120. Before incubation, three samples of each litter species were used measure the initial litter quality.

### Chemical analyses

Litter samples and initial litter were washed gently using deionized water until the water was transparent, and then oven-dried at 60 °C to constant weight (1 week) before measuring dry weight (accuracy to 0.01 g). All litter samples were ground to a powder and passed through a 0.5-mm mesh screen for quality analysis. Initial litter samples were analyzed to determine organic C, N, P, cellulose, and lignin concentrations; incubated litter samples were analyzed to determine N and P concentrations; plant powder samples were analyzed to determine the concentrations of organic C, N, P, and essential oils. Organic C concentration was analyzed using the H_2_SO_4_–K_2_Cr_2_O_7_ heat method, and N and P were quantified using Kjeldahl digestion followed by colorimetric analysis. Cellulose and lignin concentrations were determined by hydrolysis (10% H_2_SO_4_ for cellulose, 72% H_2_SO_4_ for lignin) followed by Na_2_S_2_O_3_ titration^31^. The essential oil concentration was determined by distillation^32^.

Fungi play a fundamental role in litter decomposition in wetland environments^33^, as confirmed in our previous experiments on the same litter species from the same wetlands^26^. The fungal biomass, which represents decomposer activity, can be estimated by measuring the ergosterol concentration^31^. For these analyses, the litter samples were frozen at −30 °C after sampling. The ergosterol concentration was determined by high-performance liquid chromatography^31^. For analyses, the litter material was lyophilized and ground, and then extracted in alkaline methanol at 80 °C. Solid-phase extraction through C18 cartridges was used for purification. Dry, unprocessed litters were used for blank values. These materials were stored dry at room temperature. We calculated the fungal biomass at the end of incubation using the conversion factor of 5.5 mg ergosterol g^−1^ fungal biomass^26,31^, and the values are expressed as mg g^−1^ dry weight (DW).

### Data analysis

The properties of soil, litter, and powder were compared by one-way ANOVA with species or types as the main factors.

The decomposition rate (*k*) for each litter species was calculated using equation (1):

1 $$-\,k=\,\mathrm{ln}({W}_{t}/{W}_{{0}})/t$$

where *W*_0_ is the initial litter mass and *W*_*t*_ is the mass remaining at *t* days^34^. Litter mass and remaining nutrients were calculated as percentages of initial values. The remaining litter mass was compared using three-way ANOVA with litter species, time, and treatment as the main factors. Within each litter species, the response variables were compared using two-way ANOVA with treatment and time as the main factors to test the treatment effect. Fungal biomass was compared by two-way ANOVA, with litter species and treatment as the main factors. Values were log-transformed to homogenize the variances if necessary. All statistical analyses were performed using the statistical software SPSS 21.
